# Mental Health and well-being During the COVID-19 Pandemic and After the Russian Invasion of Ukraine

**DOI:** 10.1007/s10900-023-01273-x

**Published:** 2023-08-25

**Authors:** Iuliia Pavlova, Aleksandra M. Rogowska, Stephen X. Zhang

**Affiliations:** 1 Theory and Methods of Physical Culture Department, Lviv State University of Physical Culture, Lviv, Ukraine; 2 Institute of Psychology, University of Opole, Opole, Poland; 3 Faculty of Arts, Law and Economics, Adelaide Business School, University of Adelaide, Business, Adelaide, Australia

**Keywords:** Anxiety, Depression, Life Satisfaction, Network Analysis, Perceived Stress, The Russian Invasion of Ukraine

## Abstract

The study compared the mental health of university students during the COVID-19 pandemic and the Russian invasion of Ukraine. A repeated online cross-sectional study was performed twice among university students in Ukraine: in November 2020 (Pre-war sample, *n* = 752) and September-October 2022 (During-war sample, *n* = 862). The survey measured life satisfaction (SWLS), perceived stress (PSS-10), anxiety (GAD-7), and depression (PHQ-9). Stress, anxiety, and life satisfaction levels were similar during the war and pandemic, while symptoms of depression decreased during the war, compared to the pandemic. Network analysis showed that anxiety has a crucial effect on depression and stress, and stress is most closely related to life satisfaction. The association of life satisfaction with anxiety and depression disappeared in wartime, in contrast to the pandemic. The priority of prevention and intervention programs in wartime Ukraine should focus on anxiety as the most influential factor in other mental health and well-being problems. The results showed high resistance and adaptation to war conditions among university students in Ukraine.

## Introduction

The study among university students from four Ukrainian universities showed that psycho-emotional well-being deteriorated during the COVID-19 pandemic [1]. Our previous study showed that the prevalence of mental health issues among university students from Ukraine in the first pandemic wave (May–June 2020) was 52.30% for high perceived stress symptoms, 22.90% for moderate to severe anxiety symptoms, and 29.00% for moderate to severe depression symptoms [2]. Compared to other countries (Colombia, Czechia, Germany, Israel, Poland, Russia, Slovenia, and Turkey), university students from Ukraine presented relatively lower levels (below the mean for nine countries) of perceived stress, anxiety, and depression [3]. Also, 40% of university students from Ukraine were dissatisfied with their life (Satisfaction with Life Scale scores ≤ 20). The frequencies of low life satisfaction in the other nine countries ranged from 18.9% in Colombia to 71.94% in Turkey, so the Ukrainian sample was a little below the mean for the total sample (39.46%) [4].

During the second wave of the COVID-19 pandemic, 37.65% of university students from Ukraine experienced symptoms of COVID-19 infection, 17.77% were tested for COVID-19, 1.51% were hospitalized for COVID-19, 14.46% were in strict quarantine for at least 14 days, 47.59% of their friends or relatives were infected, 7.83% reported that their friends or relatives died due to COVID-19, 23.80% lose a job because of COVID-19, and 70.48% experienced worsened economic status due to the pandemic [5]. A comparison of Polish and Ukrainian students indicate that Ukrainians report better physical health and life satisfaction while having lower symptoms of COVID-19-related PTSD, anxiety, and depression, than their Polish counterparts [6]. In addition, university students from Ukraine perceived better the positive effect of the lockdown than Polish participants. A path model showed that stress negatively affects life satisfaction directly and indirectly through increased anxiety and depression levels. Anxiety is positively associated with life satisfaction but can indirectly worsen life satisfaction by the mediating effect of depression. Depression is also directly and negatively related to satisfaction with life among university students. However, the positive association between anxiety and life satisfaction requires further explanation.

Meta-analyses showed that war impacts mental health, increasing the risk of PTSD, anxiety, and depression [7, 8]. In particular, people exposed to armed conflict on the first front lines of war are at risk of increasing psychological problems [8]. Pavlova et al. [9] demonstrated that many Ukrainian combatants showed symptoms of anxiety, depression, and insomnia. However, mental health differed depending on closeness to the war area or direct involvement. The study among Ukrainian adults showed that every second respondent presented symptoms of psychological distress (52.7%), anxiety (54.1%), and depression (46.8%) [10]. Insomnia was found in 12.1% of participants. Mental health problems were associated with some demographic variables, like gender, living in an urban area, having children or elderly persons in the household, and living in an area occupied by Russian forces.

One of the biggest challenges for the healthcare system during the war is the migration of people from the east to central and western regions of Ukraine or other countries. Research performed in 2016 among a large sample of internally displaced persons in Ukraine showed a high prevalence of PTSD (32%), depression (22%), and anxiety (17%) [11]. A more recent study performed during the current war in eastern Ukraine–Zaporizhia and Kharkiv regions among internally displaced persons and military veterans indicated that they experience high psychological stress, depression, anxiety, and intrusive memories due to war and isolation [12]. In particular, an elevated prevalence of PTSD and somatic symptoms, anxiety, depression, and sleep disturbance among internally displaced persons and Ukrainian refugees [13–16]. Hodes [17] suggests that the effect of exposure to cumulative traumatic events and losses leads to an exceptionally high risk of psychological distress.

A further study among students and personnel of four universities in war-involved regions of Ukraine showed that deterioration of psycho-emotional status was reported in 97.8% of respondents [18]. However, the prevalence of mental health problems may depend on Ukraine’s geographical region. Osokina et al. [19] showed that anxiety, depression, and post-traumatic stress disorder (PTSD) were more likely in adolescents exposed to war (Donetsk in the east of Ukraine) than those living outside the war-affected region in Ukraine (Kirovograd in central Ukraine). In particular, students from East Ukraine were four times more likely to have PTSD and three times more likely to demonstrate anxiety and depression than those from Central Ukraine.

### The Present Study

The cumulative effects of the COVID-19 pandemic, the Russian invasion of Ukraine and displacement since 2014, and the current war escalation successively increase uncertainty and instability, leading to adverse mental health outcomes in Ukrainians [17, 20–23]. Unfortunately, the healthcare system in Ukraine was already under threat in pre-pandemic times [11] but was severely damaged during the COVID-19 pandemic and the current war [21, 23–25]. The Russian invasion started simultaneously as COVID-19 cases were striking an all-time high. Due to weakening prevention strategies, loosened COVID-19 restrictions, and a low number of vaccinated people, the rate of people at higher risk for severe complications of COVID-19 and death increased dramatically in 2022. The reduced access to health services and supplies during the cumulative impact of the COVID-19 pandemic and the Russian war also contributed to an increased risk of mental health disorders [21, 26].

Considering the damaged healthcare system in Ukraine during the war, targeted interventions and preventative measures are in high demand to assess mental health and well-being. Although the war affects the psychological well-being and mental health of Ukrainians [9–19], to the best of our knowledge, the differences in the mental health of Ukrainians between the period of the pre-war COVID-19 pandemic and the during-war period have not been yet explored. The war started when the COVID-19 pandemic had not finished, so the people were already stressed and exhausted by the last challenge. In addition, the Ukrainian population is affected by media and disinformation, which may contribute to increasing distress, regardless of the degree of involvement in hostilities.

The present study will compare the perceived stress, anxiety, depression, and life satisfaction of university students from the western area of Ukraine, between two cohorts surveyed in various periods: during the second wave of the COVID-19 pandemic (Pre-war sample), and in the Autumn of 2022, in the seventh to eighth month of fighting against the Russian invaders (During-war sample). We analyze life satisfaction as a criterion for successful adaptation and resilience in the context of risk and threat. In addition, the association between life satisfaction, perceived stress, and depression will be examined using network analysis (NA) to find the critical variables for mental health during the war. Furthermore, priorities need to be established for psychotherapy, which has limited options for treating all people needing psychological support. Knowing the most critical mental health dimension will allow us to prepare selective therapy programs for target groups. To date, similar studies have not been published. Therefore, this study is exploratory, and we do not assume directional hypotheses.

## Methods

### Study Design and Procedure

The repeated cross-sectional study was performed twice in two samples of Ukrainian university students. A Pre-war sample was surveyed during the second wave of the COVID-19 pandemic (between 2 and 26 November 2020), and a During-war sample was collected during the Russian invasion of Ukraine between 26 September and 16 October 2022. Students’ trade unions and student organizations in Ukraine helped to invite university students to participate in the surveys online using a Google Forms survey. In addition, the invitation was distributed via Facebook groups, Viber groups, and Telegram channels. The survey contained information about the research and an informed consent form, assuring the anonymity and confidentiality of the survey and that participants may terminate their involvement at any time.

The study consisted of a conventional sample of students living in the Lviv and Ternopil regions. The inclusion criteria for both cohorts (Pre-war and During-war samples) were: to be at least 16 years old, and to be a student of Lviv State University of Physical Culture, Lviv Polytechnic National University, or Ternopil Volodymyr Hnatiuk National Pedagogical University. We used a quota sample to ensure the Pre-war and During-war cohorts were comparable regarding crucial demographic variables such as gender, relationship status, and study type. The Pre-war sample contained 752 individuals, and the During-war sample included 862 university students for an overall response rate of 57.6%.

The Stringency Index (SI) is a composite measure based on thirteen policy response indicators, including testing policies, face coverings, contact tracing, travel bans, school and workplace closures, and vaccination [27]. The SI ranges from 0 to 100, and 100 refers to the strictest policies in the country. According to this measure in Ukraine, SI = 60.30 (the mean number of new cases of COVID-19 daily was 211,527, and the mean number of deaths per day was 183) during the data collection in the Pre-war sample, while SI = 13.89 (the mean number of new cases of COVID-19 daily was 218, and the mean number of deaths per day was 9) when the During-war sample was surveyed [28].

### Measures

#### Demographics

The sociodemographic questions included age (years), gender (Women, Men), relationship status (Single, Coupled), faculty of study (open question), field of study (open question), study grade (1–6 study year), and the type of enrollment (Full-time, Part-time). There is also a question on participants’ mental health history: “Have you ever been diagnosed, treated, and/or monitored for any health problems listed in the table below (anxiety, depression, or post-traumatic stress disorder)? Tick the appropriate box if the answer is YES. Answer YES only if a doctor or licensed professional (e.g., psychologist) told you that you have or have had this condition. Mark “NO” if your answer is negative.“

#### Life Satisfaction

The Satisfaction With Life Scale (SWLS) of Diener et al. [29] is used to assess the global cognitive aspect of subjective well-being with five items by a seven-point response scale (from *Strongly disagree* = 1 to *Strongly agree* = 7). A higher score (range 5–35) indicates a high level of life satisfaction. The cut-off is 20 to separate people dissatisfied with their life (scores 5–19) from those satisfied (scores 20–35). The reliability in this study was Cronbach’s α = 0.83.

#### Perceived Stress

The Perceived Stress Scale (PSS-10) was developed by Cohen et al. [30] to measure how much a given life event is considered a stressful situation. The scale consists of ten items, with a five-point Likert scale of responses (from *Never* = 0 to *Very often* = 4). Higher scores (range 0–40) represent high perceived stress. The result is categorized as “No stress symptoms” (scores 0–23) and “Stress symptoms” (24–40). The reliability in the study was Cronbach’s α = 0.83.

#### Anxiety

The seven-item Generalized Anxiety Disorder (GAD-7) scale was developed by Spitzer et al. [31] to screen anxiety symptoms during the last two weeks. A four-point Likert scale of responses reflects the frequency of a given experience (*Not at all* = 0, *Nearly every day* = 3). Higher scores mean more severe anxiety symptoms (ranging from 0 to 21). Scores are categorized as “No anxiety symptoms” (scores 0–9) and “Anxiety symptoms” (scores 10–21). The reliability in this study was Cronbach’s α = 0.90.

#### Depression

The nine-item Patient Health Questionnaire (PHQ-9), developed by Kroenke et al. [32], assessed depression symptoms. Participant rate on a four-point Likert scale (*Not at all* = 0, *Nearly every day* = 3) how frequent symptoms were presented during the last two weeks. A high score indicates more severe depression symptoms (ranging from 0 to 27). We categorized the total score into “No depression symptoms” (scores 0–9) and “Depression symptoms” (scores 10–27). In this study, reliability was Cronbach’s α = 0.88.

### Participant Characteristics

The two study samples included 1614 undergraduates from Ukraine, aged between 16 and 39 (*M* = 18.52, *SD* = 1.67), including 336 men (20.82%) and 1278 women (79.18%). Among the students, one person was 16 (0.06%), while 364 people were 17 years old (22.55%). Participants studied at the Bachelor level in various faculties. The total sample consisted of a Pre-war sample (*n* = 752) and a During-war sample (*n* = 862), without differences in sample size between these groups, χ^2^(1) = 3.75, *p* = 0.053, φ = 0.001. University students represented the following universities: Lviv State University of Physical Culture (*n* = 641; including Pre-war *n* = 313, During-war *n* = 328), Lviv Polytechnic National University *(n* = 34; including Pre-war *n* = 21, During-war *n* = 13), and Ternopil Volodymyr Hnatiuk National Pedagogical University (*n* = 939; including Pre-war *n* = 418, During-war *n* = 521). Undergraduates differed in age between the Pre-war sample by about half a year (Range 17–24, *M* = 18.80, *SD* = 1.25) and During-war sample (Range 16–39, *M* = 18.27, *SD* = 1.94), *t*(1612) = 6,48, *p* < 0.001, Cohen’s *d* = 0.32), likely due to the fact there were more senior students in the Pre-war sample (Table 1). In addition, significantly more students of the Pre-war sample declared a professional clinical diagnosis of depression (25% vs. 10%), anxiety (22% vs. 7%), and PTSD (16% vs. 3%) compared to their counterparts from the During-war sample (Table 1), possibly due to the difficulty in seeing doctors or getting a diagnosis during the war. No statistically significant differences were found between Pre-war and During-war samples in gender, relationship status, and study type, as shown in Table 1.

**Table 1:** Demographic characteristics of Ukrainian university students in the Pre-war sample (during the second wave of the COVID-19 pandemic) and During-war sample (during the Russian invasion of Ukraine)

Variable	Categories	Pre-war sample (*n* = 752)		During-war sample (*n* = 862)		Total (*N* = 1614)		χ^2^	*df*	*p*	φ
		*n*	%	*n*	%	*n*	%				
Gender	Man	156	20.75	180	20.88	336	20.82	0.01	1	0.946	–0.002
	Woman	596	79.26	682	79.12	1278	79.18				
Relationship status	Single	383	50.93	445	51.62	828	51.30	0.08	1	0.781	–0.007
	Coupled	369	49.07	417	48.38	786	48.70				
Study year	First	146	19.42	305	35.38	451	27.94	90.53	3	< 0.001	0.237_#_
	Second	311	41.36	386	44.78	697	43.19				
	Third	191	25.40	105	12.18	296	18.34				
	Fourth	104	13.83	66	7.66	170	10.53				
Study type	Full-time	9	1.20	17	1.97	26	1.61	1.52	1	0.217	–0.031
	Part-time	743	98.80	845	98.03	1588	98.39				
Depression diagnosis	No	562	74.73	773	89.68	1335	82.71	62.71	1	< 0.001	–0.197
	Yes	190	25.27	89	10.33	279	17.29				
Anxiety diagnosis	No	585	77.79	802	93.04	1387	85.94	77.25	1	< 0.001	–0.219
	Yes	167	22.21	60	6.96	227	14.06				
PTSD diagnosis	No	631	83.91	839	97.33	1470	91.08	89.04	1	< 0.001	–0.235
	Yes	121	16.09	23	2.67	144	8.92				

*Note.* Percentages are accounted for within columns for each contingency table. # = effect size was assessed using Cramer’s V

### Statistical Analysis

Pearson’s χ^2^ test of independence was performed in the study to compare the demographic characteristics of the Pre-war and During-war samples. Four dependent variables are considered to be bicategorical variables (0 = *no symptoms*, 1 = *symptoms*): life dissatisfaction (SWLS), perceived stress (PSS-10), anxiety (GAD-7), and depression (PHQ-9) symptoms. The effect size was assessed using the φ coefficient for bicategorical variables and Cramer’s *V* for multicategories (i.e., study year and study level). The independent samples Student’s *t*-test was performed to compare the Pre-war with the During-war samples regarding age, life satisfaction, perceived stress, anxiety, and depression symptoms, treated as continuous variables. Cohen’s *d* coefficient was used to assess effect size.

The associations between life satisfaction perceived stress, anxiety, and depression will also be examined using linear correlation (Pearson’s *r*) and regression analysis for life satisfaction as a dependent variable and mental health dimensions as predictors. Network analysis (NA) will be performed to find a specific pattern of associations between variables. The model of associations was assessed using the extended Bayesian information criteria with the graphical least absolute shrinkage and selection operator (EBICglasso) as an estimator. Normalized centrality measures were also implemented to the NA for the total sample and in comparison of the Pre-war sample with the During-war sample. It is important to note that associations are visualized as thicker or thinner lines between nodes to indicate the magnitude between nodes. In addition, blue indicates a positive association, while orange represents negative relation between nodes (variables). To examine variables’ relevance and role in the network, we tested such centrality indices as betweenness, closeness, degree, and the expected influence. All statistical tests were performed using the JASP ver. 0.16.1.0. software for Windows.

## Results

### A Comparison of Mental Health and Well-Being Between Two Cohorts

**Fig. 1:**
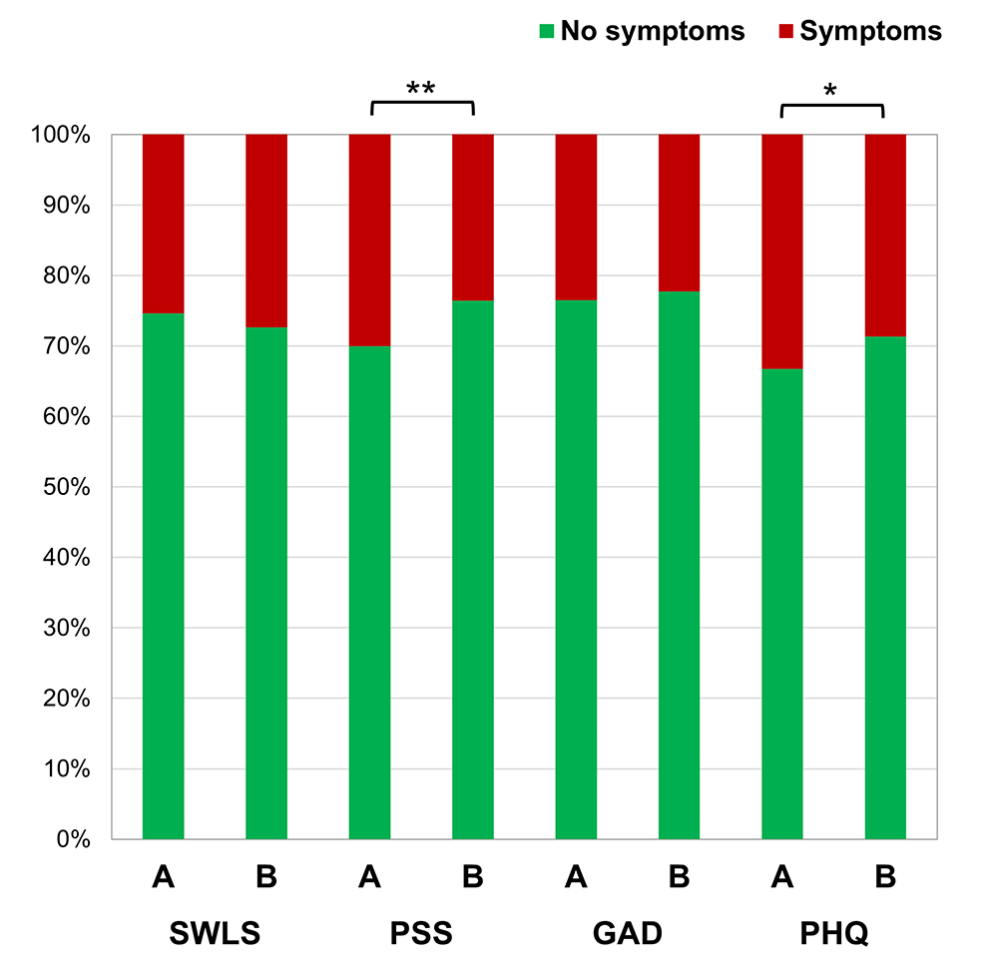
Differences between the pre-war sample **(A)** and the during-war sample **(B)** in frequencies of life dissatisfaction (SWLS), perceived stress (PSS), anxiety (GAD), and depression (PHQ) symptoms. *p < 0.05, **p < 0.01

Pearson’s χ^2^ test of independence was performed to compare the Pre-war sample with the During-war sample in terms of life dissatisfaction, perceived stress, anxiety, and depression symptoms. All variables were bicategorical using established cut-off criteria. The results are in Table 2; Fig. 1. The Pre-war sample did not differ from the During-war sample in life dissatisfaction and anxiety symptoms, but significant differences were found in symptoms of perceived stress and depression. In addition, the Pre-war sample (during the second wave of the COVID-19 pandemic) presented more symptoms of stress and depression than the During-war sample (during the Russian invasion).

**Table 2:** The Pearson’s χ^2^ test of independence for comparison Pre-war sample with the During-war sample in frequencies of life dissatisfaction, perceived stress, anxiety, and depression symptoms

Variable	Symptoms	Pre-war sample (*n* = 752)		During-war sample (*n* = 862)		χ^2^(1)	*p*	φ
		*n*	%	*n*	%			
Life dissatisfaction	No	561	74.60	626	72.62	0.81	0.369	0.02
	Yes	191	25.40	236	27.38			
Perceived stress	No	526	69.95	659	76.45	8.70	0.003	–0.07
	Yes	226	30.05	203	23.55			
Anxiety	No	575	76.46	670	77.73	0.36	0.547	–0.02
	Yes	177	23.54	192	22.27			
Depression	No	502	66.76	615	71.35	3.97	0.046	–0.05
	Yes	250	33.25	247	28.65			

*Note*. Percentages are accounted for within columns for each contingency table

The statistical analysis was performed again for life satisfaction, perceived stress, and symptoms of anxiety and depression, but all these variables were considered continuous (Table 3). The independent samples Student’s t-test showed that the Pre-war sample (n = 752) did not differ significantly from the During-war sample (n = 862) in life satisfaction, perceived stress, anxiety, and depression symptoms.

**Table 3:** The independent samples Student’s *t*-test to compare the pre-war sample (students surveyed during the second wave of the COVID-19 pandemic in 2020) with the during-war sample (students surveyed during the Russian invasion in 2022)

Variable	Pre-war sample		During-war sample		*t*(1612)	*p*	*Cohen’s d*
	(*n* = 752)		(*n* = 862)				
	*M*	*SD*	*M*	*SD*			
Life satisfaction	23.29	5.73	22.89	6.02	1.73	0.168	0.07
Perceived stress	19.68	6.96	19.16	6.35	1.38	0.117	0.08
Anxiety	6.33	4.82	6.43	4.71	1.57	0.674	–0.02
Depression	7.78	5.69	7.30	5.41	–0.42	0.084	0.09

### Associations of Life Satisfaction with Perceived Stress and Symptoms of Anxiety and Depression

**Fig. 2:**
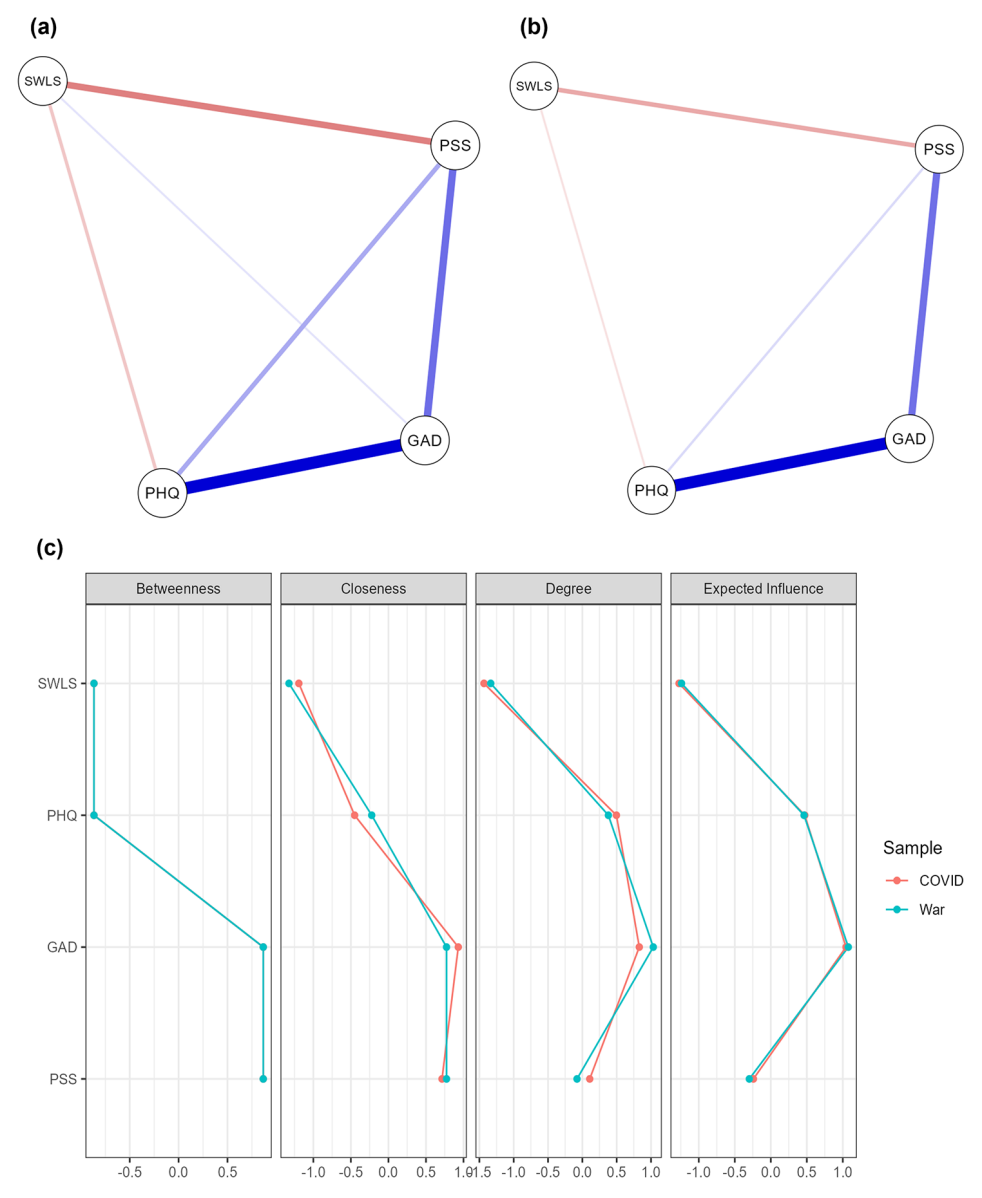
Association between life satisfaction perceived stress, anxiety, and depression in Ukrainian university students: **(a)** network analysis in the pre-war sample (second wave of the COVID-19 pandemic), **(b)** network analysis in the during-war sample (Russian invasion), **(c)** Centrality plot for assessment of associations between the variables in the network analysis. SWLS = satisfaction with life scale (life satisfaction), PSS = Perceived Stress Scale (to assess stress symptoms), GAD = General Anxiety Disorder (anxiety symptoms), PHQ = Patient Health Questionnaire (depression symptoms). Blue links represent a positive association, but orange links have a negative relation between nodes

A network analysis was performed to find the pattern of associations between all variables (life satisfaction, perceived stress, anxiety, and depression) and how they influenced each other. As shown in Fig. 2a,b, depression is the strongest related to anxiety (positive association). A weaker positive association is found between anxiety and stress. The positive association between stress and depression is even more vulnerable in the Pre-war sample, while it disappears in the During-war sample. Life satisfaction is negatively related to both stress (stronger association) and depression (weaker link), but in the During-war sample, these relationships are weaker. A poor positive connection between life satisfaction and anxiety in the Pre-war sample is observed. In contrast, life satisfaction is unrelated to anxiety and depression among undergraduates from the During-war sample. We performed an additional analysis, using betweenness, closeness, degree of strength, and expected influence, to deeply test the associations in the NA model (Fig. 2c).

Betweenness can be defined as the degree of connectivity, assessed by the number of times a node is part of all pairs of related nodes in the network. Stress and anxiety are interrelated positively, while life satisfaction and depression are interconnected negatively (Fig. 2c). In closeness, a closer location to other nodes is shown in the more central nodes in the network. Life satisfaction is the farthest from anxiety and stress but closer to depression. The degree of strength is assessed here as the sum of all the paths that connect the nodes in terms of the mean of correlation weights. Figure 2c shows that life satisfaction is related to the following variables (from the strongest to the weakest): stress, depression, and anxiety. Finally, the expected influence represents the essential variables that can act as a bridge between the adjacent nodes. The order of bridge variables in the present study is as follows: (1) anxiety, (2) depression, (3) stress, and (4) life satisfaction (Fig. 2c).

## Discussion

### A Comparison of Mental Health Between Pre-War and During-War Samples

The research aimed to compare life satisfaction, perceived stress, anxiety, and depression symptoms in two cohorts of Ukrainian university students: during the war and one year before the Russian invasion of Ukraine, during the second wave COVID-19 pandemic. The study revealed that university students presented significantly fewer symptoms of stress and depression during the war than during the second wave of the COVID-19 pandemic. In particular, life dissatisfaction was reported by 25.40% of students before the war and 27.38% during the war. Among students, 30.05% experienced high perceived stress during the second wave of the COVID-19 pandemic and 23.55% during the war. Moderate or severe anxiety symptoms were declared in 23.54% of the Pre-war sample and 22.27% of the During-war sample. Depression risk was found in 33.25% of university students before the war and 28.65% of their counterparts during the war. However, no differences were found between the two cohorts when variables were considered continuous.

Compared to the previous study [10], much fewer university students than Ukrainian adults reported symptoms of high stress (52.7%), anxiety (54.1%), and depression (46.8%). However, Xu et al. [10]performed their study in March 2022, at an early stage of the war, whereas the present study was conducted more than half a year later. During several months, people may have adapted to war and developed resilience strategies to cope with stress. Therefore, the levels of psychological distress (stress, anxiety, and depression) were similar during the war to those during the COVID-19 pandemic but likely lower than those from the beginning of the war. We assumed that at the beginning of the war, the level of distress was extremely high but gradually decreased in areas that were not front-line.

It is also important to note that the current research was performed in the Western regions of Ukraine, which are classified as relatively safe territories. At the same time, the war affected the entire territory, and the western regions were bombarded, while air alarms warning of the possibility of missile attacks were regular. In the first three weeks of the war, the entire territory of the country was subjected to heavy shelling, and a total of 1,200 rockets were launched for bombing [33]. From the beginning of the war to the end of our study, air alarms sounded 28,394 times, including 378 times in the Lviv region and 409 in the Ternopil region total of 55 rocket hits were recorded in these regions [34]. Accordingly, all respondents were in the territory of hostilities and threatening circumstances. With the beginning of the war, universities stopped the educational process and announced vacations for two weeks. Early attestation and graduation of cadets receiving higher military education was carried out. The educational process in Ukrainian higher education institutions began to resume in mid-March in distance or mixed formats, and unique study conditions were provided for those students who joined the Armed Forces of Ukraine or territorial defense units or engaged in volunteer activities. Opportunities were created for all students to continue their studies, but studying in conditions of constant uncertainty, low level of security, lack of shelters, and frequent air alerts harmed the psycho-emotional state. Therefore, the levels of distress may be lower than in the previous study [10], which was performed among Ukrainians around the whole country. However, the study among adolescents indicated that those from war-affected regions of Ukraine (Donetsk) have higher levels of PTSD, anxiety, and depression than their counterparts from regions not exposed directly to war (Kirovograd) [19].

Furthermore, other studies showed [8, 9] that the highest risk of PTSD, anxiety, and depression is presented in people exposed to armed conflict and those who must migrate because of war [11–16]. On the other hand, this part of the country has received the largest number of refugees. As of March 2022, the number of internally displaced persons in the territory of the Lviv region alone was about 400,000. As of the beginning of 2023, more than 245,000 internally displaced persons are officially registered in the Unified Information Database of Ukraine in the Lviv region and about 100,000 in the Ternopil region. In addition, the cumulative effects of home loss, separation from loved ones, and loneliness may contribute to the deterioration of mental health and well-being [17]. It is worth noting that the research was conducted in particularly active regions.

Cross-cultural research showed that university students from Ukraine reported fewer symptoms of stress, PTSD, anxiety, depression, and life dissatisfaction than those from other countries, including Poland, Slovenia, Turkey, Israel, Colombia, or Germany [2–6]. Ukrainian students seem to adapt well to difficulties and are more resilient than university students from many other countries. The healthy competencies and the hope to win the war could contribute to low levels of distress among university students from Ukraine during the war.

### Network Analysis

The second aim of this study was to find the mental health dimension that will be the most important for treating the current war-related crisis. The cumulative effects of the COVID-19 pandemic and the Russian invasion of Ukraine caused Ukraine’s healthcare system to collapse [21, 23–25]. Research indicates that many people who require psychological support are out of the mental health care system [11, 26]. Therefore, prioritizing health care is critical with these severely limited resources. The NA analysis showed that the vital variable contributing to mental health is anxiety, which affects stress and depression, leading to a deterioration of life satisfaction.

The current study aligns with our previous findings [6], which indicated similar associations between life satisfaction, stress, anxiety, and depression. However, this study extends our previous research by evidencing that anxiety should be treated to the greatest extent in campus prevention and intervention programs. The other prevention and intervention strategies should focus on decreasing stress and depression. As indicated in our study, stress should be treated to increase life satisfaction levels. Depression is primarily associated with high anxiety levels and, to a lesser degree, stress.

Interestingly, although a poor positive connection was presented between life satisfaction and anxiety during the second wave of the COVID-19 pandemic, no association between these variables was found during the war. The relationship between anxiety and life satisfaction is fully mediated by stress and depression during the war. Likely, higher anxiety levels helped maintain numerous restrictions during the pandemic, but it was not more useful during the war. Further studies should be focused on explaining the meaning of anxiety for life satisfaction during various disasters and crises.

### Limitations of the Study

Although the study was performed on a large sample of university students, some limitations prevent generalizations. First, the study was performed in Western Ukraine, so the results can not adequately represent regions where there are contact fights and people are held captive by the occupier. At the same time, the obtained results can be used for one-fourth of Ukraine’s regions, which are similar from the point of view of involvement in hostilities. Secondly, the study was performed in three universities, so the results cannot be generalized to all of the academic community. The sample included undergraduates, so little is known if the results would be the same among those enrolled in postgraduate study. Women also prevailed over men in our samples, which could affect the results.

Further study should be performed in all regions of Ukraine, with a more representative sample of university students, including various types of universities (e.g., medical, technical, art), study levels, and gender. Moreover, comparing the mental health of students from Ukraine with those from other countries could shed more light on the well-being problem from a broader perspective, depending on culture, economic situation, geographical location, or regional specificity. Furthermore, the two cohorts were not equal regarding the clinical mental health diagnosis. We controlled both samples for clinical diagnosis of PTSD, anxiety, and depression from a professional psychiatrist or psychologist. All three mental health disorders were diagnosed more frequently among Pre-war participants than those of the During-war group. Although the clinical diagnosis was more difficult due to the subsequent deterioration of the health care system of Ukraine, these differences could contribute to the study’s results.

## Conclusions

University students’ mental health and well-being during the war do not change significantly from the second wave of the COVID-19 pandemic. However, since the trauma of war could last many years after the war finished, the mental health and well-being levels should be monitored systematically among Ukrainians of various generations. Current prevention and intervention strategies should be prioritized to decrease anxiety first and then to reduce stress and depression. Chaaya et al. [21] recommend educating Ukrainians on self-recognition of the symptoms of mental health disorders, ensuring the provision of essential services, increasing screening for mental illness in emergency and primary care facilities, increasing the possibility of providing psychological support by professionals, and using telepsychiatry to fill the gaps created in health care through the COVID-19 pandemic and war. Various psychological therapies are needed to improve or maintain the well-being of populations in Ukraine, as suggested by scientists [11, 22, 35]. Since the healthcare system of Ukraine is broken, non-governmental organizations (NGOs), doctors, and all the front-line workers worldwide should extend their humanitarian support to the Ukrainian population, as suggested by Uwishema et al. [24]. Kalaitzaki et al. [23] call for help from professional associations, voluntary organizations, and people worldwide to work collaboratively to address the war-affected populations’ mental health needs before it is too late.

## Data Availability

The data that support the findings of this study are available from the corresponding author upon request.

## References

[1] Pavlenko, V., Kurapov, A., Drozdov, A., Korchakova, N., Reznik, A., & Isralowitz, R. (2022). Mental Health and Substance Use among Ukrainian “Help Profession” students during the COVID-19 pandemic. *International Journal of Mental Health and Addiction*, 1–4. 10.1007/S11469-022-00831-Z/METRICS. 10.1007/s11469-022-00831-z PMC9063613 35531309

[2] Cuero-Acosta, Y. A. (2021). Mental health prevalence and predictors among university students in nine countries during the COVID-19 pandemic: a cross-national study. *Scientific Reports 2021 11:1*, *11*(1), 1–13. 10.1038/s41598-021-97697-3. 10.1038/s41598-021-97697-3 PMC8452732 34545120

[3] *Journal of Clinical Medicine*, *10*(13), 2882. 10.3390/JCM10132882/S1.

[4] *BMC Public Health*, *21*(1), 1–19. 10.1186/S12889-021-12288-1/TABLES/4.

[5] Çınar, O. (2021). Exposure to COVID-19 during the First and the Second Wave of the Pandemic and Coronavirus-Related PTSD Risk among University Students from Six Countries—A Repeated Cross-Sectional Study. *Journal of Clinical Medicine 2021, Vol. 10, Page 5564*, *10*(23), 5564. 10.3390/JCM10235564. 10.3390/jcm10235564 PMC8658425 34884266

[6] Rogowska AM, Kuśnierz C, Pavlova I, Chilicka K (2022). A path model for Subjective Well-Being during the Second Wave of the COVID-19 pandemic: A comparative study among polish and ukrainian University students. Journal of Clinical Medicine.

[7] Morina N, Stam K, Pollet TV, Priebe S (2018). Prevalence of depression and posttraumatic stress disorder in adult civilian survivors of war who stay in war-afflicted regions. A systematic review and meta-analysis of epidemiological studies. Journal of Affective Disorders.

[8] Franco OH (2022). Mental health of migrants with pre-migration exposure to armed conflict: A systematic review and meta-analysis. The Lancet Public Health.

[9] Pavlova I, Graf-Vlachy L, Petrytsa P, Wang S, Zhang SX (2022). Early evidence on the mental health of ukrainian civilian and professional combatants during the russian invasion. European Psychiatry.

[10] Xu, W., Pavlova, I., Chen, X., Petrytsa, P., Graf-Vlachy, L., & Zhang, S. X. (2023). Mental health symptoms and coping strategies among Ukrainians during the Russia-Ukraine war in March 2022. 10.1177/00207640221143919. 10.1177/00207640221143919 36598090

[11] Roberts B, Makhashvili N, Javakhishvili J, Karachevskyy A, Kharchenko N, Shpiker M, Richardson E (2019). Mental health care utilisation among internally displaced persons in Ukraine: Results from a nation-wide survey. Epidemiology and Psychiatric Sciences.

[12] *American Journal of Orthopsychiatry*. 10.1037/ORT0000537.

[13] Johnson RJ, Antonaccio O, Botchkovar E, Hobfoll SE (2022). War trauma and PTSD in Ukraine’s civilian population: Comparing urban-dwelling to internally displaced persons. Social Psychiatry and Psychiatric Epidemiology.

[14] Rizzi, D., Ciuffo, G., Sandoli, G., Mangiagalli, M., de Angelis, P., Scavuzzo, G.,… Ionio, C. (2022). Running Away from the War in Ukraine: The Impact on Mental Health of Internally Displaced Persons (IDPs) and Refugees in Transit in Poland. *International Journal of Environmental Research and Public Health 2022, Vol. 19, Page 16439*, *19*(24), 16439. 10.3390/IJERPH192416439. 10.3390/ijerph192416439 PMC9778520 36554321

[15] Cheung A, Makhashvili N, Javakhishvili J, Karachevsky A, Kharchenko N, Shpiker M, Roberts B (2019). Patterns of somatic distress among internally displaced persons in Ukraine: Analysis of a cross-sectional survey. Social Psychiatry and Psychiatric Epidemiology.

[16] Konstantinov V, Reznik A, Isralowitz R (2022). The impact of the russian–ukrainian War and Relocation on Civilian Refugees. Journal of Loss and Trauma.

[17] Hodes M (2022). Thinking about young refugees’ mental health following the russian invasion of Ukraine in 2022. Clinical Child Psychology and Psychiatry.

[18] Kurapov A, Pavlenko V, Drozdov A, Bezliudna V, Reznik A, Isralowitz R (2022). Toward an understanding of the russian-ukrainian War Impact on University Students and Personnel. Journal of Loss and Trauma.

[19] Osokina O, Silwal S, Bohdanova T, Hodes M, Sourander A, Skokauskas N (2022). Impact of the russian Invasion on Mental Health of Adolescents in Ukraine. Journal of the American Academy of Child and Adolescent Psychiatry.

[20] Bryant RA, Schnurr PP, Pedlar D (2022). Addressing the mental health needs of civilian combatants in Ukraine. The Lancet Psychiatry.

[21] Chaaya, C., Thambi, D., Sabuncu, V., Abedi, Ö., Osman Ahmed Osman, R., Uwishema, A., & Onyeaka, O. (2022). Ukraine – Russia crisis and its impacts on the mental health of ukrainian young people during the COVID-19 pandemic. *Annals of Medicine and Surgery*, *79*, 10.1016/J.AMSU.2022.104033. 10.1016/j.amsu.2022.104033 PMC9221679 35765517

[22] Jawaid, A., Gomolka, M., & Timmer, A. (2022). Neuroscience of trauma and the Russian invasion of Ukraine. *Nature Human Behaviour 2022 6:6*, *6*(6), 748–749. 10.1038/s41562-022-01344-4. 10.1038/s41562-022-01344-4 35437314

[23] Kalaitzaki AE, Tamiolaki A, Vintila M (2022). The compounding effect of COVID-19 and War in Ukraine on Mental Health: Α Global Time Bomb soon to explode?. Journal of Loss and Trauma.

[24] *Postgraduate Medical Journal*, *98*(1162), 569–571. 10.1136/POSTGRADMEDJ-2022-141895. 10.1136/postgradmedj-2022-141895 PMC9340026 35654572

[25] Goto R, Pinchuk I, Kolodezhny O, Pimenova N, Skokauskas N (2023). Mental health services in Ukraine during the early phases of the 2022 russian invasion. The British Journal of Psychiatry.

[26] Romaniuk P, Semigina T (2018). Ukrainian health care system and its chances for successful transition from soviet legacies 14 Economics 1402 Applied Economics. Globalization and Health.

[27] *Nature Human Behaviour 2021 5:4*, *5*(4), 529–538. 10.1038/s41562-021-01079-8. 10.1038/s41562-021-01079-8 33686204

[28] *Our World in Data*. Retrieved March 25, 2023, from https://ourworldindata.org/coronavirus.

[29] Diener E, Emmons RA, Larsem RJ, Griffin S (2010). The satisfaction with Life Scale. Journal of Personality Assessment.

[30] Cohen S, Kamarck T, Mermelstein R (1983). A global measure of perceived stress. Journal of Health and Social Behavior.

[31] Spitzer RL, Kroenke K, Williams JBW, Löwe B (2006). A brief measure for assessing generalized anxiety disorder: The GAD-7. Archives of Internal Medicine.

[32] Kroenke K, Spitzer RL, Williams JBW (2001). The PHQ-9: Validity of a brief depression severity measure. Journal of General Internal Medicine.

[33] U.S. Department of Defense (2022, March 23). Senior Defense Official Holds a Background Briefing. *Senior Defense Official*. Retrieved August 3, 2023, from https://www.defense.gov/News/Transcripts/Transcript/Article/2977137/senior-defense-official-holds-a-background-briefing/.

[34] Air Alarms. (n.d.). Statistics of air alarms in Ukraine - The whole country. Retrieved August 3 (2023). from https://air-alarms.in.ua/.

[35] Javanbakht, A. (2022). Addressing war trauma in ukrainian refugees before it is too late. *European Journal of Psychotraumatology*, *13*(2), 10.1080/20008066.2022.2104009. 10.1080/20008066.2022.2104009 PMC9359191 35959204

